# Hydrophobic pp-HMDSO Coating for Three-Dimensional Cell Culture

**DOI:** 10.3390/jfb17070334

**Published:** 2026-07-09

**Authors:** Marina Rakhmanova, Anastasia Leonteva, Maxim Chagin, Evgeniya Ermakova, David Sergeevichev, Vladimir Richter, Marina Kosinova, Anna Nushtaeva

**Affiliations:** 1 Scientific Center of Genetics and Life Sciences, Sirius University of Science and Technology, 1 Olimpiysky Ave., Sirius Federal Territory, Sirius 354340, Russia; anastleont@mail.ru (A.L.); sergeevichev.ds@talantiuspeh.ru (D.S.); 2 Institute of Chemical Biology and Fundamental Medicine Siberian Branch of the Russian Academy of Sciences, 8 Acad. Lavrentiev Ave., Novosibirsk 630090, Russia; rakhmanovamar@gmail.com (M.R.); richter@1bio.ru (V.R.); 3 Nikolaev Institute of Inorganic Chemistry Siberian Branch of the Russian Academy of Sciences, 3 Acad. Lavrentiev Ave., Novosibirsk 630090, Russia; chagin@niic.nsc.ru (M.C.); ermakova@niic.nsc.ru (E.E.); marina@niic.nsc.ru (M.K.)

**Keywords:** biomaterial surface, functional coatings, modification 3D cell culture, spheroids, pp-HMDSO, PECVD, hydrophobicity, U-87 MG, HMC3

## Abstract

The interaction of biomaterial surface with cells is a pivotal factor in tissue engineering and three-dimensional (3D) modeling. This paper presents an approach to modifying polystyrene surface by plasma-enhanced chemical vapor deposition of thin plasma-polymerized hexamethyldisiloxane (pp-HMDSO) films to enhance biocompatibility and stimulate the formation of 3D cellular structures. The coatings were characterized by SEM, EDS, XPS, FTIR, AFM, contact angle measurements, and surface free energy (SFE) analysis. A hydrophobic surface initiates 3D structure formation by ensuring uniform cell repulsion and stimulating intercellular interactions. Biological evaluation was performed on U-87 MG (glioblastoma) and HMC3 (microglia) cell lines. For U-87 MG, the pp-HMDSO layer proved critical: cell death and atypical adhesion occurred on untreated plastic, whereas stable spheroids formed on the modified surface. HMC3 cells formed small spheroids even on unmodified surfaces, but on pp-HMDSO coatings, the process was more intense and the structures more uniform due to surface hydrophobicity. These results demonstrate the potential of plasma-polymerized HMDSO films as a scalable platform for creating biomaterials with controlled properties for 3D culturing.

## 1. Introduction

In recent years, three-dimensional (3D) cell cultures have emerged as a subject of comparable significance in laboratory research to traditional suspension or adherent cell cultures [1,2]. Furthermore, tumor cells cultured under 3D conditions have been shown to more accurately mimic in vivo cell interactions [3,4]. Specifically, three-dimensional cultures are anticipated to serve as a substitute for experimental animals [5,6]. Spheroid models are widely regarded as the simplest and most reproducible 3D cultures. The methods for forming and culturing spheroids are often divided into two categories: scaffold-free methods, which include the hanging drop method, low-adhesion surfaces, and rotating bioreactors, and scaffold-based methods, which utilize biocompatible hydrogels [7]. Culture plastic with a low-adhesion surface offers a simple platform for 3D culture formation, requiring no specialized equipment or additives like magnetic particles [8,9]. A critical factor influencing the capacity of cells to aggregate and form spheroids is the nature of their interaction with the surface of the culture plastic. The initiation of spheroid formation necessitates the employment of hydrophobic or ultra-low-adhesion surfaces, a strategy that has been demonstrated to effectively mitigate cell adhesion to the substrate, thereby promoting intercellular contacts [10,11,12]. As Ferrari et al. [10] have emphasized, the formation of cell aggregates is contingent on surface hydrophobicity, and the efficient formation of spheroids necessitates coatings with maximum water-repellent properties. A critical parameter determining the wettability and adhesion properties of a surface is surface energy. The low surface energy of a material results in high contact angles and minimizes interactions with cells and proteins in the culture medium.

In the domain of biomaterials, polymeric materials for surface modification have gained significant recognition, including poly(2-hydroxyethyl methacrylate) (PHEMA), polyethylene glycol (PEG), zwitterionic polymers, and poly(2-methoxyethyl acrylate) (PMEA) [13,14,15]. However, conventional coating application methodologies (e.g., spin-coating, dip-coating, or solvent-casting) are inadequate for high-throughput and reproducible modification of standard 96-well plates. These methodologies fail to ensure uniformity and controlled coating thickness for complex well geometries. Moreover, these methodologies frequently necessitate multi-step processes involving toxic solvents and are difficult to scale up, which limits their application for obtaining samples with reproducible properties in sufficient quantities for bioscreening studies. A promising approach to creating specialized coatings is surface modification using plasma-enhanced chemical vapor deposition (PECVD) through the decomposition of organosilicon compounds, such as hexamethyldisiloxane (HMDSO) (CH_3_)_3_Si–O–Si(CH_3_)_3_. HMDSO is a stable and non-toxic monomer with high vapor pressure at room temperature. The employment of this monomer in chemical vapor deposition (CVD) processes ensures high film deposition rates and the ability to control chemical structure and functional properties by varying deposition conditions. This method facilitates the synthesis of biocompatible coatings with designated hydrophobicity, a consequence of the preservation of methyl groups (−CH_3_) on the film surface. As demonstrated in the works of Lazauskas et al. [16], plasma-polymerized HMDSO (pp-HMDSO) films exhibit the presence of methyl groups, which contribute to a low surface energy and result in superhydrophobic properties, manifesting in contact angles reaching up to 170°. The findings of other studies corroborate the conclusion that such coatings are biocompatible and do not exhibit toxicity, thereby effectively protecting cells from potentially toxic substrates [17]. It is also important to study the stability of the properties of modified surfaces for subsequent biological studies [14,15].

A recent global study found that although over 80% of researchers recognize the importance of 3D models, most do not use them routinely in their research, primarily due to the high cost of the plates [18]. Presently, commercial plates with specialized low-adhesion coatings are employed for routine research, with the majority of manufacturers opting not to disclose the composition of these coatings. A comparative study of plates from various manufacturers reveals significant variations in performance. Xing et al. [8] demonstrated that the choice of Ultra-Low Attachment (ULA) plates critically affects the formation kinetics, size, and viability of spheroids. This underscores the necessity to establish standardized and reproducible methodologies for the fabrication of surfaces that exhibit controlled hydrophobicity and reduced surface energy. In order for the creation of three-dimensional spheroids to be considered a standard technique in the field of cell biology, it is essential that the results obtained are consistently reproducible. It is imperative that the cells within each well aggregate into a single spheroid, without the formation of satellite spheroids [8,19].

This study aims to develop and characterize pp-HMDSO coatings on polystyrene (PS) for 3D spheroid culture. The coatings were characterized by FTIR, XPS, SEM, AFM, EDS, and contact angle measurements. Spheroid formation and viability were evaluated on pp-HMDSO-coated versus unmodified PS using U-87 MG and HMC3 cells. The correlation between film properties and biological responses was then analyzed.

## 2. Materials and Methods

### 2.1. Blanks for Modification

The films were deposited on three types of substrates: (1) Si(100), (2) Ge(111), and (3) polystyrene substrates.

1. The characterization of pp-HMDSO films was conducted using a semiconducting n-type Si(100) substrate (KEF-4.5) with dimensions of 10 × 10 × 0.47 mm^3^ (Russia). The pp-HMDSO/Si(100) samples were used for a range of analytical techniques, including scanning electron microscopy (SEM), Fourier transform infrared spectroscopy (FTIR), X-ray photoelectron spectroscopy (XPS), atomic force microscopy (AFM), ellipsometry, and wettability studies.
2. EDS analysis was performed on pp-HMDSO/Ge(111) structures to avoid the influence of the silicon substrate when determining the elemental composition. EDS analysis of the polystyrene substrates was not carried out.
3. For the biological experiments, eight independent fragments were cut, and all were coated with pp-HMDSO under identical plasma conditions; they are designated as Jet Biofil “pp-HMDSO” samples 1–7 (test group).

Non-treated PS 96-well plates (#TCP002096, Jet Biofil, Guangzhou, China) were used as received. Prior to plasma-enhanced chemical vapor deposition, the plates were sectioned into smaller fragments (approximately 100 × 20 × 10 mm^3^, each containing 16 wells) to fit the reactor chamber. Two types of controls were used:

(i) Untreated control—intact wells from the same plate batch with no treatment.
(ii) Air-plasma-only control—fragments exposed to air plasma under the same conditions but without HMDSO supply. These samples were prepared from the same batch of plates.

The volatile monomer precursor was hexamethyldisiloxane ((CH_3_)_3_Si)_2_O (99% purity, Macklin, Shanghai, China). Synthetic air of high purity grade (Russia) was used as the gas activator.

### 2.2. Preparation of pp-HMDSO Films

**Figure 1:**
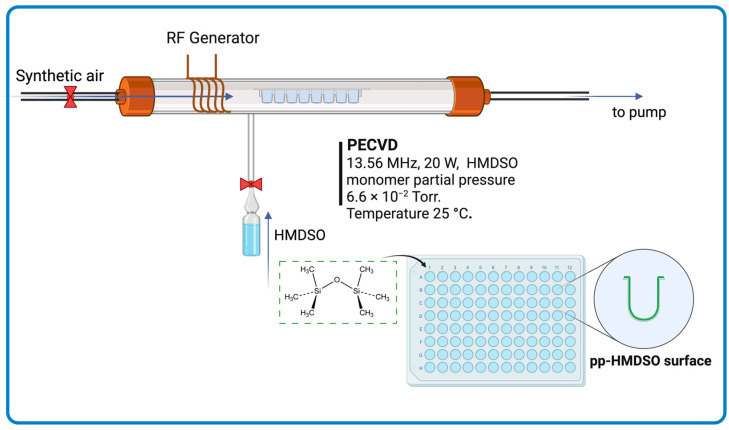
Scheme of the PECVD reactor used for pp-HMDSO deposition. RF: radio frequency (13.56 MHz). Discharge power: 20 W. Monomer partial pressure of HMDSO: 6.6 × 10^−2^ Torr. Created in BioRender. Rakhmanova, M. (2026) https://BioRender.com/1pczxku (accessed on 7 April 2026).

The plasma-enhanced chemical vapor deposition technique was utilized to synthesize pp-HMDSO thin films on Si(100) substrates (10 × 10 mm^2^) and PS plates (10 × 10 mm^2^ and 10 × 100 mm^2^). Figure 1 presents a scheme of the PECVD reactor. The configuration comprises a feedstock supply system, a horizontal quartz reactor, a reaction product collection system, a pumping system, and an RF (radio frequency) generator with a matching device and an external inductor. The HMDSO precursor was located in a quartz container at ambient temperature; the partial pressure was set by a regulating valve. Film deposition occurred at room temperature (25 °C). Prior to the film deposition process, the Si(100) substrates underwent a standard degreasing procedure and chemical etching. The plastic plates were exposed to air plasma for a duration of two minutes prior to the deposition of the film. The experimental conditions for the deposition of pp-HMDSO were as follows: a discharge frequency of 13.56 MHz, a discharge power of 20 W, and a monomer partial pressure of HMDSO of 6.6 × 10^−2^ Torr. The experimental conditions have been demonstrated to result in the formation of a film characterized by a pronounced organic quality.

### 2.3. Characterization of pp-HMDSO Films

The pp-HMDSO films were characterized on layers grown on Si(100) and PS substrates.

An ellipsometry method was employed to ascertain the film thickness (d). Multi-angle measurements were performed on a LEF-3M monochromatic ellipsometer (Priborostroitelniy Zavod, Feodosiya, Russia) with an operating wavelength of λ = 632.8 nm. The measurements were conducted at five different beam incidence angles on the sample, ranging from 50° to 70° with a 5° step. It was calculated with an error of 3%, which is determined by measuring the parameters ψ and δ, as well as the choice of the initial model of the inverse problem. The variation in thickness across a 1 cm^2^ sample did not exceed 5%. The samples of two different thicknesses were used for analysis. pp-HMDSO films with about 100 nm thickness were used for XPS, AFM, and contact angle measurements. The thick films (620 nm) were used to get clear FTIR spectra with distinguished absorption bands.

A SEM 5000 (Siqtec, Shenzhen, China) scanning electron microscope (SEM) equipped with an Oxford Xplore 30 Aztec Live Lite (Oxford Instruments, High Wycombe, UK) energy dispersive spectroscopy (EDS) attachment was utilized to study the homogeneity, surface morphology, and elemental composition of the films. The pp-HMDSO film deposited on the Ge(111) substrate was used for EDS mapping.

The FTIR absorption spectra of 620 nm pp-HMDSO films on the Si(100) substrate were recorded on a SCIMITAR FTS 2000 Fourier transform spectrometer in the wavenumber range of 4000–400 cm^−1^ with a resolution of 2 cm^−1^. The spectra were subsequently normalized to the film thickness.

Roughness analysis of the samples was performed using an Ntegra Prima II atomic force microscope (NT-MDT, Moscow, Russia) in tapping mode. The MFM01 probe, with the following parameters, was utilized: probe length 225 µm, width 35 µm, thickness 2.5 µm, force constant 3 N/m, resonant frequency 70 kHz. The scanning parameters that were utilized are listed in the following: setpoint 5; gain 0.3; rate 0.5–1.0; point 256; scanning time 4–8 min. To determine the RMS value, a 5 µm × 5 µm area of the sample was analyzed. To confirm the obtained result, three such areas were examined.

The chemical composition of the sample surfaces was studied using a photoelectron spectrometer from SPECS Surface Nano Analysis GmbH (Berlin, Germany). The spectrometer was equipped with a PHOIBOS-150 hemispherical analyzer and an XR-50 characteristic X-ray source with a double Al/Mg anode. The recording of the spectra was accomplished through the utilization of non-monochromatic Al K_α_ radiation (hβ = 1486.6 eV). The relative concentrations of elements in the analysis zone were determined based on the integrated intensities of the XPS lines, taking into account the photoionization cross sections of the corresponding terms [20]. To facilitate a comprehensive analysis, the spectra were decomposed into their constituent components. Subsequent to background subtraction through the implementation of the Shirley method, the experimental curve underwent decomposition into a series of lines corresponding to the photoemission of electrons from atoms in disparate chemical environments. The data processing was executed using the CasaXPS package, version 2.3.25 (Casa Software Ltd., Dewon, UK) [21]. The peak shape was approximated by a symmetric function obtained by multiplying the Gaussian and Lorentzian functions. To account for the charging effect, the carbon C *1s* peak (E_b_ = 284.80 eV), corresponding to carbon in sp^2^ hybridization (amorphous carbon), was utilized.

Contact-angle experiments are performed using the Contact Angle System (OCA Data Physics, Stuttgart, Germany). The measurement of contact angle (CA) was performed using deionized water and diiodomethane (98% purity, Russia). The contact angle values were calculated as the average of 3–4 measurements. The free surface energy (E_s_, mN/m) and its polar and dispersion components (E_s_^p^ and E_s_^d^) were estimated using three different models: Neumann, Owens–Wendt, and Wu. This calculation was contingent upon the contact angles and surface tension values of two probe liquids (water and diethylene glycol) of contrasting polarity.

### 2.4. Cell Lines

The cell lines HMC3 (#C0005019, AddexBio, San Diego, CA, USA) and U-87 MG (ATCC #HTB-14, Manassas, VA, USA) were procured from the American Type Culture Collection (ATCC, Manassas, VA, USA) and utilized for the spheroid formation. The absence of mycoplasma contamination in all cell lines was confirmed through RT-PCR analysis (Biolabmix, Novosibirsk, Russia). The genetic identification of the cell lines was performed using the GOrDIS Plus kit (GORDIZ, Moscow, Russia). The STR profiles correspond to those published in the ATCC, DSMZ, and Cellosaurus international databases.

The U-87 MG cells were cultivated in Minimum Essential Medium α (α-MEM) (#12571063, Gibco™, New York, NY, USA), while the HMC3 cells were cultivated in Dulbecco’s Modified Eagle Medium/Nutrient Mixture F-12 (DMEM/F12) (#42400028, Gibco™, New York, NY, USA). The culture media for 2D culture contained 10% fetal bovine serum (FBS) (#A316040, Thermo Fisher, Waltham, MA, USA), 1X GlutaMAX™ solution (L-alanyl-glutamine 200 mM in 0.85% NaCl) (#35050061, Gibco™, New York, NY, USA), and 1X antibiotic-antimycotic solution (100 U/mL penicillin, 0.1 ng/mL streptomycin, and 0.25 μg/mL amphotericin) (#15140122, Gibco™, Waltham, MA, USA).

The cells were detached from the substrate using TripLE™ (Gibco BRL Co., Invitrogen, Waltham, MA, USA) upon reaching a monolayer. The detached cells were then diluted with a complete growth medium at a ratio of 1:3 to 1:4 (by volume) for the purpose of further culturing. All cell cultures were cultivated at 37 °C in a humidified atmosphere of 5.0 ± 0.5% CO_2_ in a Binder incubator (Binder, Ulm, Germany).

### 2.5. Spheroid Formation

Spheroids were formed using the liquid layering method in 96-well plates. A thorough analysis was conducted on the outcomes of three independent experiments. To form spheroids, cells were washed with 2 mL of 1X phosphate-buffered saline (PBS) and detached from the substrate using TrypLE™ (#12604013, Gibco, Invitrogen) for 5 min at 37 °C. Subsequently, the TrypLE™ solution was inactivated by the addition of 1 mL of complete medium. The cell count was determined by utilizing a LUNA-II™ cell counter (Logos Biosystems, Anyang-si, Republic of Korea). The requisite number of cells (2500 or 5000) were seeded into the wells of uncoated, air-plasma-treated (JET Biofil, Guangzhou, China) or low-adhesion coated U-shaped plates for 3D culture (96-well Nunclon™ Sphera™ U-shaped-bottom plates (#174925, Thermo Scientific, Waltham, MA, USA), 96-well 3D Sphearo™ Ultra-low Adsorption Surface (#TCP130096, JET Biofil, Guangzhou, China), along with experimental samples (number 1–7) at a total volume of 100 µL in culture serum-free medium (3D medium). The cultivation of cells was conducted over a period of five days.

Serum-free media containing bovine serum albumin (BSA) or similar protein supplements help stabilize the aggregates of cells [22,23]. Research undertaken several decades ago demonstrated that the utilization of either DMEM in isolation or in conjunction with F12 medium, with various combinations of growth factors, steroids, and hormones, constitutes an effective methodology for the in vitro culturing of neurons and glial cells, as well as the generation of spheroids [22,24]. The 3D spheroid formation medium was prepared using a formulation of DMEM/F12, to which the following components were added: 1X GlutaMAX™ Supplement (#35050061, Gibco™, New York, NY, USA), 1X Antibiotic-Antimycotic (#15240062, Gibco™, New York, NY, USA), 20 ng/mL EGF (Epidermal Growth Factor; #E9644, Sigma-Aldrich, Burlington, MA, USA), 20 ng/mL fibroblast growth factor basic (bFGF; #PHG0261, Gibco™, New York, NY, USA), 5 µg/mL insulin (#I9278, Sigma-Aldrich, Burlington, MA, USA), and 2% B27 Plus Supplement (#A35828010, Gibco™, Burlington, MA, USA). The following reagents were utilized: 1) 4% Albumin Bovine Serum (BSA, #126593, Sigma-Aldrich, Burlington, MA, USA). On the third day of the culture period, an additional 100 µL of the 3D medium was introduced into each well. Spheroids were cultivated at 37 °C in a humidified atmosphere containing 5.0 ± 0.5% CO_2_, within a Binder incubator (Binder, Germany).

### 2.6. Spheroid Analysis

Microscopic analysis of spheroid formation was performed using an Evos M5000 microscope (Thermo Fisher Scientific, Waltham, MA, USA). The resulting images were then subjected to analysis using Fiji (ImageJ 2.16.0/1/54p, Java 1.8.0_442 (64-bit)) software on days 1, 3, and 5 of culture. A thorough analysis was conducted on the outcomes of three independent experiments.

#### 2.6.1. Calculation of the Spheroid Volumes

Spheroid volumes were assessed using light microscopy (Nikon Eclipse Ti-S) and Fiji (ImageJ) software. The volume of the spheroid was calculated using the following formula: V = 0.5 × L × W^2^, where L is the diameter that connects the pair of outermost points on the spheroid contour, and W is the largest diameter perpendicular to L [25]. In instances where multiple spheroids were formed in a single well, the spheroid with the greatest volume was utilized.

#### 2.6.2. Live/Dead Staining

On the fifth day, the viability of the 3D models was assessed in accordance with the protocol outlined in [26], with certain modifications. To this end, fluorescein diacetate (FDA) (#F1303, Thermo Fisher, USA), propidium iodide (PI) (#556463, BD Bio-sciences, Franklin Lakes, NJ, USA), and Hoechst 33342 (#H1399, Invitrogen, USA) were utilized to stain live cells, dead cells, and the total number of cells, respectively. FDA is a non-fluorescent molecule that is converted into fluorescent fluorescein by intracellular esterases present in viable cells with intact membranes. Consequently, only live cells exhibit green fluorescence upon FDA staining. PI (propidium iodide) is a membrane-impermeant dye that can only enter cells with compromised membranes, i.e., dead or dying cells. Hoechst 33342 is a DNA-binding dye that passively diffuses across cell membranes, allowing it to stain all nuclei (both live and dead) without the need for membrane disruption.

In this experiment, 100 microliters of the medium was extracted from each well. Then, 100 microliters of FDA (final concentration: 10 μg/mL) was diluted in DMEM/F12, without additional additives. The mixture was then incubated under standard conditions for 20 min. Subsequently, 100 μL of the sample was collected once more. Then, 100 μL of PI (5 μg/mL) and Hoechst (2.5 μg/mL), both diluted in PBS, were added and incubated under standard conditions for 10 min. Subsequently, the spheroids were washed with phosphate-buffered saline (PBS), transferred to a microscopy plate, and imaged using an EVOS M5000 microscope. In instances where spheroids and/or cells adhered to the plastic surface, resulting in difficulty transferring the models to the microscopy plate, additional photographs were taken in a U-shaped plate. Subsequently, all resulting images were combined into a single image. The images were then subjected to analysis using Fiji (ImageJ).

### 2.7. Statistical Data Processing

Statistical analysis was performed using GraphPad Prism version 10.3 (GraphPad Software, San Diego, CA, USA). Two-way ANOVA followed by Tukey’s post hoc test was used for multiple comparisons of spheroid volumes. Data are presented as the mean ± standard error of the mean. Quantitative analysis of spheroid number and volume was performed only on day 5 using three independent wells per condition. For the time-course images (days 1, 3, and 5), the same wells were photographed to visualise spheroid development; no statistical analysis was applied to these sequential images. For each cell line, eight independent pp-HMDSO-coated PS substrates (sample 1–8) were prepared, each with three technical replicate wells (24 independent replicates per plate). The data from all fragments were pooled for the final quantitative analysis (day 5).

## 3. Results

### 3.1. Characterization pp-HMDSO Films

**Figure 2:**
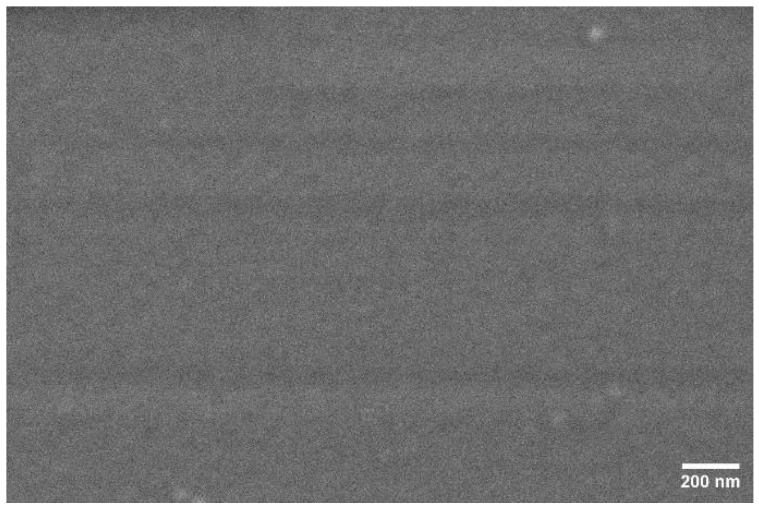
The SEM image of the surface of the pp-HMDSO film.

Figure 2 presents the SEM image of the surface of the pp-HMDSO film grown on a Si(100) substrate. The image clearly demonstrates that the film surface is characterized by smoothness, uniformity, and the absence of defects and pores. It exhibits good morphological homogeneity, which provides effective coverage.

**Figure 3:**
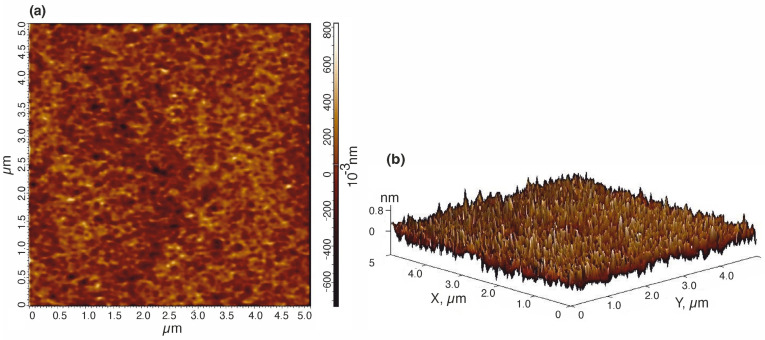
AFM images of the pp-HMDSO film. The (**a**) two- and (**b**) three-dimensional AFM topography images of the pp-HMDSO film surface.

The two- and three-dimensional AFM images of the pp-HMDSO film grown on Si(100) substrate are displayed in Figure 3. The absence of inhomogeneities on the film surface and high smoothness are also confirmed by AFM. The mean surface roughness (RMS) of the films was determined to be 1.5 nm (5 × 5 µm^2^ scanning area), as calculated from the AFM data. Figure S1 shows AFM images of the surface of the uncoated PS and the pp-HMDSO film deposited on the PS substrate; RMS values were 4.52 and 4.36 nm, respectively.

**Figure 4:**
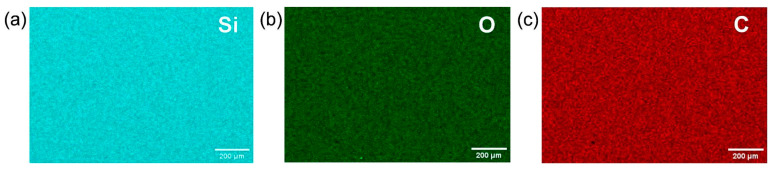
EDS elemental mapping images of pp-HMDSO film. (**a**) silicon; (**b**) oxygen; (**c**) carbon.

The pp-HMDSO films demonstrate adequate uniformity in their elemental composition as well. This assertion is corroborated by the EDS mapping data, as illustrated in Figure 4. It is evident that the distribution of silicon, oxygen, and carbon on the film surface is relatively uniform.

The analysis of FTIR spectra is an invaluable tool for comprehending the diverse types of bonds present in pp-HMDSO films. The interpretation of the FTIR spectra is based on literature data [27,28,29].

**Figure 5:**
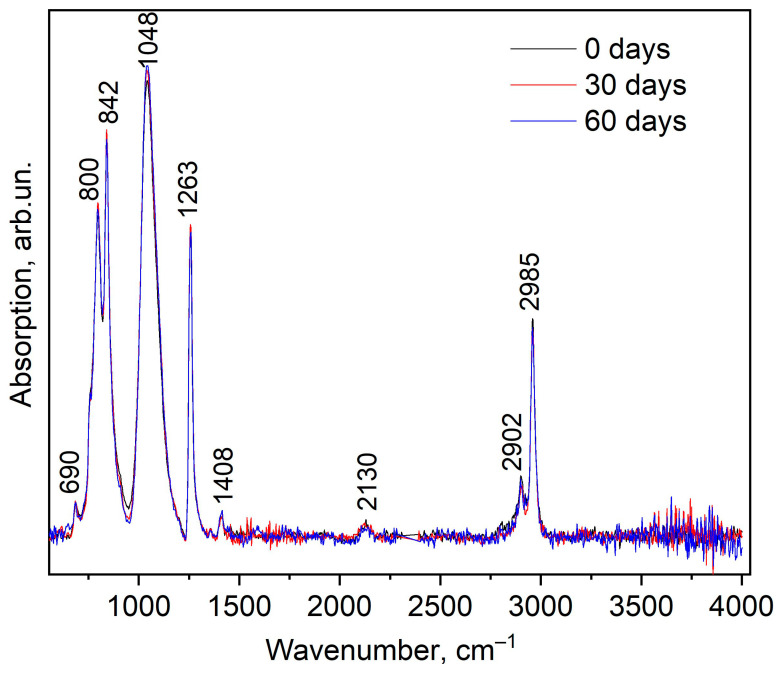
FTIR spectra of as-grown and aged pp-HMDSO film. Black line—as-grown film; blue line—after 30 days of the air storage; red line— after 60 days of the air storage. The pp-HMDSO coating shows good stability over 60 days, as indicated by the absence of changes in the intensity and shape of the characteristic bands. Arb. un. stands for arbitrary unit.

Figure 5 presents FTIR spectra of the as-grown and aging pp-HMDSO film. The FTIR spectra show the main Si–O–Si stretching band at 1048 cm^−1^. Bands characteristic of methyl groups attached to silicon appear at 2985, 2902, 1263, and 842 cm^−1^, which are assigned to CH_3_ stretching, symmetric bending, and rocking modes, respectively (Table 1). The band at 2130 cm^−1^ corresponds to Si–H, indicating partial fragmentation of the HMDSO precursor in the plasma. These data confirm the organo-rich structure of the pp-HMDSO film. Figure 5 demonstrates the stability of the pp-HMDSO film during 60 days of storage. The absence of changes in the intensity and shape of the bands associated with the Si-CH_3_ group indicates the persistence of the functional properties of the film. A representative single spectrum of the as-grown pp-HMDSO film is shown in Figure S2.

**Figure 6:**
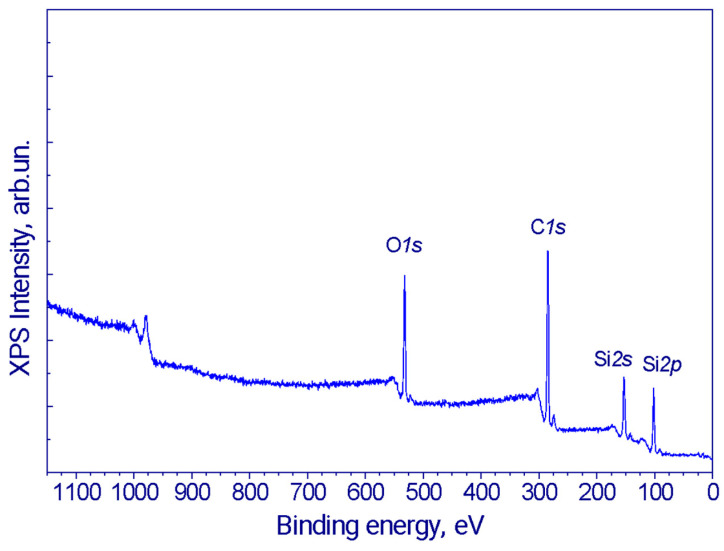
Survey XPS spectrum of pp-HMDSO film samples. Arb. un. stands for arbitrary unit.

The survey spectrum of the studied sample is shown in Figure 6. The XPS spectrum revealed peaks corresponding to the elements Si, C, and O, with no indication of other elemental impurities. The peak at 975 eV corresponds to the O KLL line. The relative atomic concentrations of the elements, as determined based on the XPS data, are presented in Table 2.

The calculated elemental ratios provide information about the chemical structure of the pp-HMDSO film. The [O]/[Si] ratio of 0.65 is much lower than the stoichiometric value for fully inorganic SiO_2_ (2.0), indicating incomplete oxidation and the retention of a significant organic component. The [C]/[Si] ratio of 2.34 reflects the high carbon content arising from methyl groups (Si–CH_3_) incorporated into the film. The low [O]/[C] ratio (0.28) further confirms the organic-rich, polymer-like nature of the coating. These chemical characteristics are consistent with the hydrophobic surface required for spheroid formation.

**Figure 7:**
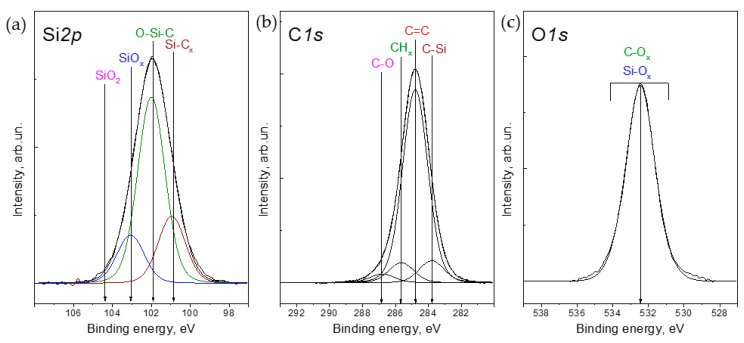
High-resolution XPS spectra of the pp-HMDSO film. (**a**) Si *2p*, (**b**) C *1s*, and (**c**) O *1s* spectra of the pp-HMDSO sample. The spectra are normalized to the integrated intensity of the Si *2p* spectrum. Arb. un. stands for arbitrary unit.

The detailed spectra of Si *2p*, C *1s*, and O *1s* are presented in Figure 7a. The Si *2p* spectrum displays three peaks with binding energies in the range of 101.0, 101.9, and 103.0 eV (Table 3). The first peak is attributed to the formation of bonds between silicon atoms and carbon atoms [30,31,32]. The maximum peak observed in the region of 101.9 eV is attributed to silicon forming an O–Si–C bond. Indeed, in the extant literature, the observed binding energy of this peak for analogous films is in the region of 101.9 eV [33,34]. The peak observed at approximately 103.0 eV is attributable to silicon bonding with oxygen and carbon, accompanied by a comparatively elevated oxygen “content” [33,34]. A thorough examination of the available data reveals that no feature corresponds to SiO_2_ (E_b_ = 104.2 eV).

Figure 7b presents the C *1s* spectra of the studied sample. The C *1s* spectra are characterized by the presence of multiple peaks, indicative of carbon in diverse environments (Table 4). For instance, the peak near 284.8 eV, corresponding to amorphous carbon, was utilized as an internal standard to account for the effect of sample charging. A closer look reveals that only two low-intensity peaks are observed in the region of high binding energies, near 285.7 and 286.8 eV, which are assigned to C–H and C–O, respectively. Additionally, a maximum of approximately 283.8 eV is detected in the spectra, which is ascribed to the formation of a bond between carbon and silicon within the SiCOH film composition.

As illustrated in Figure 7c, the O *1s* spectrum of the examined film is presented. The O *1s* spectrum is described by a single symmetrical peak with a binding energy of approximately 532.5 eV, which is consistent with oxygen present in the SiCOH film structure and in the surface C-O_x_ groups.

The most significant parameters influencing the performance of a material in biomedical applications are surface free energy, the presence of functional groups, and surface roughness. The contribution of the various components to the surface free energy (SFE) was determined by contact angle measurements using two probe liquids: distilled water and diiodomethane. The water contact angles of the pp-HMDSO film deposited on Si(100) and PS substrates were 109.5 ± 0.8° and 96 ± 3°, respectively (Table 5), indicating a hydrophobic character. These values are close to those reported for polydimethylsiloxane films (106–108°) [35,36] and pp-SiOCH films [37]. Diiodomethane contact angles on PS substrates were not determined due to the lack of solvent resistance of PS (swelling/dissolution).

The total surface free energy, including its dispersion (E_s_^d^) and polar (E_s_^p^) components, is summarized in Table 6. The surface exhibited a low SFE of 22.74 and 26.59 mN/m (Neumann method) for the pp-HMDSO film deposited on Si(100) and PS substrates, respectively.

Thus, the plasma-chemical decomposition of hexamethyldisiloxane at room temperature yielded amorphous hydrogenated pp-HMDSO films. The concentrations of Si, C, and O elements, as determined by XPS analysis, were found to be 25.1 at.%, 58.6 at.%, and 16.3 at.%, respectively.

### 3.2. Evaluation of the Coating Effect on Spheroid Formation

**Figure 8:**
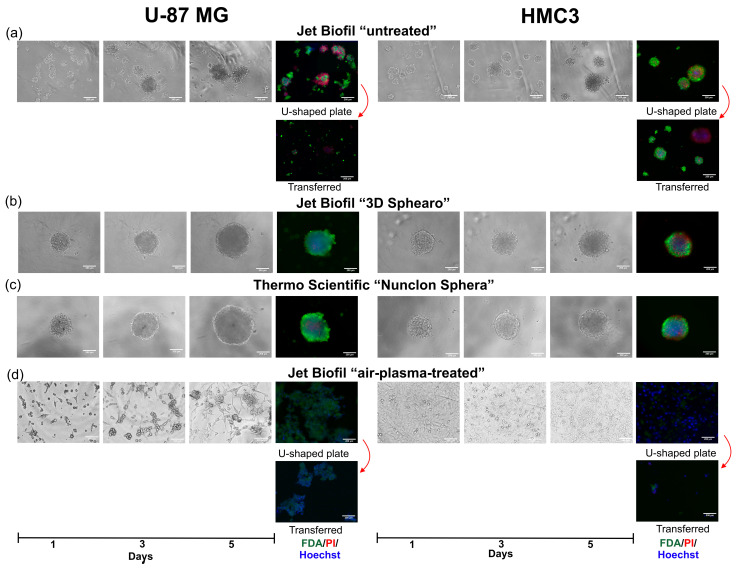
A comparative analysis of the formation and effectiveness of spheroidal models on control samples of ULA plastic: (**a**) negative control: untreated polystyrene (JET Biofil untreated); (**b**,**c**) positive commercial controls: (**b**) 3D Sphearo™ Ultra-low Adsorption Surface (JETBiofil), (**c**) Nunclon™ Sphera™ (Thermo Fisher Scientific); (**d**) additional control to assess the effect of the plasma itself: treated by air plasma only (air-plasma-treated, JETBiofil). The series of images below the graph show the growth of the spheroids from days 1 to 5 (scale bar = 200 μm), and cell viability within these spheroids was assessed on day 5. The green signal indicates viable cells, the red signal indicates dead cells, and the blue signal indicates the total cell number. Following the transfer process, the spheroids were relocated to a microscopy plate, while the U-shaped plate contained samples that had not undergone transfer. Light and fluorescence microscopy were utilized. Scale bar 200 μm.

The quality of the experimental coating on the culture plate for 3D model formation was evaluated. The ability of U-87 MG and HMC3 cells to form spheroids was assessed, as well as the volume and viability of the models on day 5 of culture. U-87 MG is a human glioblastoma cell line, and HMC3 is a microglial cell line isolated from the human brain. First, commercially available low-adhesion 96-well plates, which enable the production of stable spheroid models, were analyzed. Figure S3 presents biological replicates of spheroids formed on the first day of cultivation. Commercial plastic coated with 3D Sphearo™ Ultra-low Adsorption Surface (JETBiofil, Guangzhou, China) and Nunclon™ Sphera™ (Thermo Fisher Scientific), as well as uncoated plastic (untreated, JETBiofil), were utilized as negative controls (Figure 8 and Figure S3). As a supplementary control, a plastic (untreated, JETBiofil) was also utilized in the creation of experimental plates that were coated with air plasma only (Figure 8d and Figure S3d).

For U-87 MG cells on uncoated plastic (JETBiofil untreated, Figure 8a and Figure S3), day 1 cultures showed small, loose aggregates of rounded cells and random attachment of individual cells to the surface. These aggregates later merged, forming multiple loose spheroids. Transferring the spheroids from the U-shaped culture plate to the microscopy plate after viability staining proved difficult. This was likely due to the attachment of aggregates and spheroids to the plastic surface. Upon attempted transfer (Figure 8), the spheroids dissociated into single cells and small clusters, indicating weak intercellular adhesion. Consequently, the viability of U-87 MG spheroids was lower than that in the control plates and on the coated experimental plates. For HMC3 cells, small spheroids initially formed, which then merged and increased in size through cell proliferation. By day 5, several large spheroids had formed. Notably, spheroids developed not only at the bottom of the U-shaped wells but also along the well edges. These peripheral spheroids exhibited reduced viability compared with those at the well bottom. Nevertheless, the overall viability of HMC3 spheroids was comparable to that of spheroids grown on coated plates.

The culture plastic was coated by air plasma only, a process which resulted in the promotion of cell adhesion to the surface of the plate. In the case of U87MG cells, the formation of small cell aggregates was also observed. Following the process of cell transfer, it was observed that cells exhibited low adherence to one another and to the surface of the plate (Figure 8d and Figure S3d). HMC3 cells were distributed uniformly across the surface of the plastic, without forming any observable spheroidal structures.

On 3D Sphearo™ and Nunclon™ Sphera™ control plates, a single spheroid formed in the center of the well for both U-87 MG and HMC3 cells within the first day, which then increased in size. On the fifth day of culture, U-87 MG spheroids were dominated by viable (FDA+) cells, with a small number of dead (PI+) cells, while HMC3 models exhibited viable spheroid formation, with a large core of dead (PI+) cells. In commercial 96-well plates, cell suspensions aggregated into a single spheroid in the vast majority of cases (>90%), so the objective was to achieve this “gold standard” [8,19].

**Figure 9:**
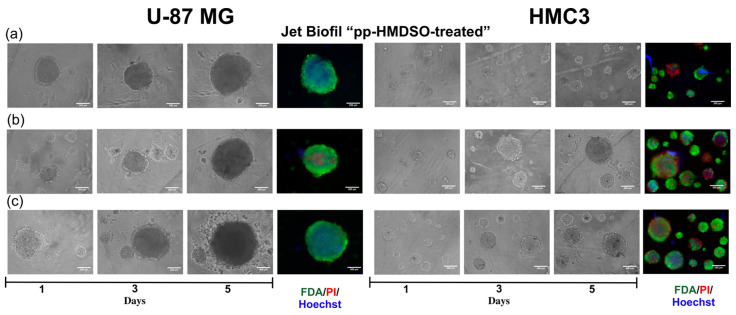
An investigation into the efficacy of an experimental coating during the formation of a spheroidal model. Sample plate with pp-HMDSO: (**a**) sample 1; (**b**) sample 2; (**c**) sample 3. The formation of spheroids from 2500 U-87 MG and HMC3 cells was observed on days 1, 3, and 5 of cultivation, and cell viability within these spheroids was assessed on day 5. The green signal indicates viable cells, the red signal indicates dead cells, and the blue signal indicates the total cell number. Following the transfer process, the spheroids were relocated to a microscopy plate, while the U-shaped plate contained samples that had not undergone transfer. Light and fluorescence microscopy were utilized. Scale bar 200 μm.

Examples of spheroid formation on the first day of cultivation, depending on the seeding density (2500 or 5000 cells/well) on experimental pp-HMDSO-coated plastic plates, in three biological replicates, are shown in Figure S4. For all experimental pp-HMDSO-coated plastic plates, one or more large spheroids of U-87 MG cells formed on the first day of culture, followed by fusion, compaction, and growth of the spheroids over the following days. For samples 2 (Figure 9b) and 3 (5000 U-87 MG cells, Figure S4c), cell aggregates with partial cell attachment were observed on day 1. However, by the fifth day, the formation of large spheroids, devoid of any indications of adhesion, was also observed. It is important to note that no significant decrease in cell viability was observed in comparison to the control group. Furthermore, the material did not demonstrate acute toxicity under these experimental conditions. As demonstrated in Figure S5, the efficacy of sample coating in promoting spheroid formation is further evidenced by additional examples using pp-HMDSO-coated plastic plates.

The viability of U-87 MG models formed on the pp-HMDSO-coated plastic plates was comparable to that of the models on the coated control plates (Figure 8, Figure 9 and Figure S3). The number of U-87 MG models in the experimental samples was comparable to that of the control spheroids and significantly larger than that of models on untreated plastic (Figure 8, Figure 9 and Figure S5).

**Figure 10:**
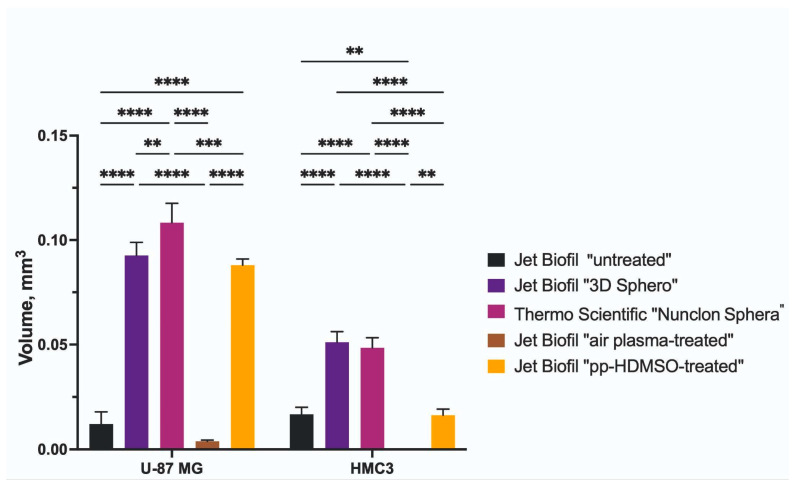
Comparison of the volumes of spheroidal models in the control and experimental plate. Black—negative control: untreated polystyrene (JET Biofil untreated); violet and purple—positive commercial controls: 3D Sphearo™ Ultra-low Adsorption Surface (JETBiofil) and Nunclon™ Sphera™ (Thermo Fisher Scientific); brown—additional control to assess the effect of the plasma itself: treated by air plasma only (air-plasma-treated, JETBiofil); orange—experimental plate pp-HMDSO-coated (JETBiofil). Number of U-87 MG and HMC3 spheroids on day 5 of culture, depending on the type of culture plate used. Data are shown as mean ± SE. Two-way ANOVA followed by Tukey’s post hoc test. Statistical significance was defined as *p* < 0.0001 (****), *p* < 0.001 (***), and *p* < 0.01 (**).

In the case of HMC3 cells, both the experimental plates and the uncoated plate demonstrated the formation of numerous small spheroids, some of which were fully viable and others completely nonviable (Figure 8). The volume of the largest spheroids in this case was more comparable to that of the uncoated control than to the volumes of spheroids in Nunclon™ Sphera™ and Biofil plates (Figure 10 and Figure S6). It is noteworthy that on the experimental plates, fusion of models occurred exclusively when they were in close proximity, suggesting the potential for inhomogeneity of the coating. The formation of multiple small spheroids by HMC3 cells could be influenced by several factors, including possible subtle variations in coating properties on the curved well walls (which are difficult to characterize directly) (Figure S7) or the intrinsic lower proliferative activity of these cells (Figure S3).

The data obtained in this study demonstrate that pp-HMDSO films combine high hydrophobicity, low surface free energy, and no apparent cytotoxicity under the tested conditions, making them a promising platform for targeted spheroid formation. The efficacy of the pp-HMDSO ULA coating was demonstrated for U-87 MG cells, which exhibit a critical reliance on low adhesion. However, for HMC3 cells, the coating efficiency was inadequate to produce single compact spheroids in each well. This phenomenon is likely attributable to coating inhomogeneity resulting from the parallel orientation of the substrate relative to the predominant precursor flow within the PECVD chamber. Subsequent efforts will center on the optimization of sample placement geometry and deposition parameters, with the objective of attaining coating versatility across a range of cell lines. Future studies will also need to include more comprehensive biocompatibility analyses (including systemic toxicity, hemocompatibility, sensitization, and preservation of cell phenotype).

## 4. Discussion

The economic burden of producing spheroids is considrably higher than that of 2D models, necessitating the use of specialized media and plastics, growth factors, hydrogels, or specific equipment [38]. A pivotal strategy for the development of such models is cell self-assembly, a process that facilitates a more precise emulation of the structural and functional characteristics of native tissues [39]. These methodologies leverage the capacity of cells to self-organize in response to physicochemical stimuli, as exemplified by scaffold-free systems that promote cell aggregation into spheroids without external support [40].

**Figure 11:**
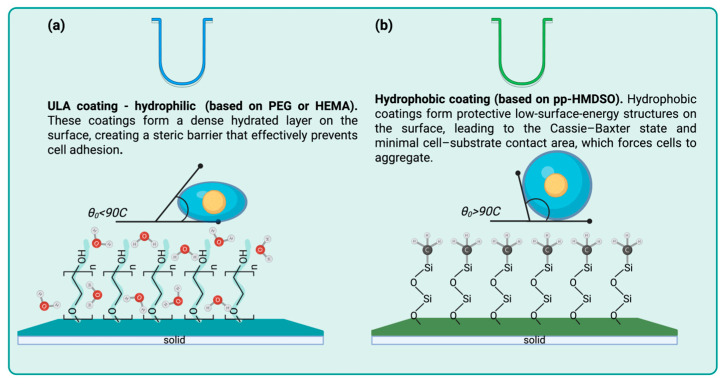
Schematic of spheroid formation on hydrophilic and hydrophobic surfaces. (**a**) ULA coating with hydrophilic layer; (**b**) hydrophobic coating pp-HMDSO. Created in BioRender. Rakhmanova, M. (2026) https://BioRender.com/rpycr54 (accessed on 7 April 2026).

The successful formation of spheroids necessitates the utilization of specialized surfaces that impede cell adhesion to the substrate. As illustrated in Figure 11, adhesion can be reduced through two distinct approaches.

1. The application of branched polymers with a high number of -OH groups, such as polyHEMA or PEG, is a specific coating method [41]. It has been established that these polymer coatings reduce cell adhesion through two mechanisms. Firstly, steric repulsion is caused by the dense packing of polymer chains on the surface. Secondly, a dense hydrated layer is formed, which creates a physical and energetic barrier preventing contact between proteins and cells and the substrate [42].

2. Alternatively, the formation of low-energy hydrophobic surfaces has been proposed (Ferrari et al. [10,11]). Hydrophobic coatings form low-surface-energy protective structures on the surface. This results in the formation of the Cassie–Baxter state, characterized by a minimal contact area between cells and the substrate. This minimal contact area is a prerequisite for the aggregation of cells.

The present study proposes a modification of polystyrene plate with a hexamethyldisiloxane coating (pp-HMDSO) and investigates its capacity to induce 3D cellular structures on two human brain cell lines (U-87 MG and HMC3). SEM and AFM analyses revealed that the pp-HMDSO films exhibited high uniformity, absence of defects, and minimal roughness (RMS 1.5 nm). The elemental composition of the samples was subsequently analyzed using EDS and XPS. These analytical methods confirmed the presence of silicon, oxygen, and carbon in the samples. The O/Si ratio of 0.65 (Table 2) indicates incomplete oxidation and preservation of the material’s organic structure, which is characteristic of plasma-polymerized siloxanes [16,17]. The FTIR spectra are dominated by the Si–O–Si bands (1048 cm^−1^) and intense peaks characteristic of methyl groups associated with silicon (2985, 2902, 1263, 842 cm^−1^), as illustrated in Figure 5. The band at 2130 cm^−1^, corresponding to Si–H, indicates partial fragmentation of the monomer in the plasma, which is observed in modes with a sufficiently high discharge energy [16]. Contact angle measurements were conducted to ascertain the surface’s hydrophobic characteristics, revealing a water contact angle of 109.5° ± 0.8° (Table 5). Additionally, the low surface free energy was determined to be 22.07 mN/m using the Owens–Wendt method (refer to Table 6 for details). These values are consistent with the literature data for films obtained from HMDSO and hydrophobic siloxane materials [35,36,37]. As emphasized in the works of Ferrari et al. [10,11,12], the low surface free energy of the substrate is the key factor weakening the adhesion of cells to the substrate and stimulating their aggregation into spheroids. The uniform repulsion of cells from such a surface appears to act as a trigger for compaction, which is consistent with the concept of the need for maximum water-repellent ability for the successful formation of 3D structures.

Typically, non-adhesive coatings or substrates with a certain degree of wettability promote the formation of spheroids, where cell clustering occurs under the influence of gravity or shear stress. In such conditions, the activation of intercellular interaction mechanisms is observed, accompanied by the expression of molecules such as E-cadherin, which contributes to a compact cell structure, and the inhibition of caspase-mediated cell death [43]. Precise testing on cell lines has demonstrated that the presence of a coating is imperative for U-87 MG cells. When cells were cultured on unmodified PS, a decrease in cell viability, non-specific adhesion to the substrate, and the absence of the formation of stable three-dimensional aggregates were observed (Figure 8 and Figure S3). Treating the culture plastic with air plasma alone promoted cell adhesion to the plate surface. On a surface with a deposited pp-HMDSO film, the formation of compact spheroids was observed, which were comparable in size and viability to spheroids obtained using commercial ULA plates (Nunclon™ Sphera™, JET Biofil) (Figure 9 and Figures S4 and S5). The developed coating has been shown to reproduce the conditions necessary for initiating the formation of 3D cellular structures in tumor cell cultures with high proliferative activity. However, for HMC3 microglial cells, the result was less clear. The formation of numerous small cellular aggregates was observed in experimental samples and untreated plastic. Some of these aggregates were nonviable, and their sizes were smaller than those of commercial analogs.

It can be posited that this phenomenon is not entirely attributable to the imperfection of the coating on the modified plates themselves. Rather, it is also plausible that fundamental differences in the biology of the two lines are a contributing factor. The U-87 MG cell line exhibits high levels of proliferation and aggregation; even on a non-uniformly hydrophobic surface, they rapidly form a single compact spheroid due to active cell division and fusion [44]. The formation of a compact spheroid is contingent upon the presence of molecules that ensure tight junctions between cells. U-87 MG expresses cadherins (e.g., cadherin-22, further induced under hypoxic conditions), which promote rapid and efficient aggregation [44,45]. The HMC3 line is an immortalized line of microglia, which are resident macrophages of the central nervous system. Their biological characteristics are fundamentally different from those of tumor cells [42,46].

Microglia, a normal cell line, do not exhibit a tendency to aggregate [47]. The formation of stable three-dimensional structures by microglia frequently necessitates signals from other cells, including glioblastoma cells [48]. The weak adhesion characteristics of these cells under serum-free conditions likely resulted in insufficient cell-to-cell adhesion, thereby hindering the formation of a large spheroid and leading to the formation of multiple small aggregates [44]. Furthermore, previous studies have demonstrated that HMC3 significantly increases the levels of collagens, collagen receptors (ITGB1/ITGA3, DDR2), and modulators of cell-collagen adhesion, such as TGFβ-induced protein (TGFBI) [48,49]. This phenomenon may also elucidate the observed absence of single spheroid formation and the subsequent decline in cell viability, attributable to the compaction of the spheroid model. This compaction results in constrained oxygen access and diminished cell proliferative activity. The process of obtaining single homogeneous spheroids from such cells necessitates a heightened degree of control over the hydrophobicity and homogeneity of the applied coating, in addition to the utilization of a combination of different coating precursors.

Despite their widespread use, commercial ULA plates exhibit variability in efficiency among manufacturers, and the composition of these plates is often not disclosed [8,9]. In contrast, the developed pp-HMDSO coating facilitates the fabrication of hydrophobic surfaces with known chemistry and low surface energy. Additionally, plasma deposition is compatible with the intricate geometry of 96-well plates and does not necessitate the use of toxic solvents, a crucial consideration for scaling and bioscreening studies [13]. The primary constraint of the present study is the inadequate efficiency of spheroid formation induction on the current coating for HMC3 cells. An additional source of variability may be attributed to the heterogeneity of deposition, which arises from the orientation of substrates relative to the precursor flow within the PECVD chamber.

Subsequent efforts will concentrate on optimizing deposition conditions, including modifying the spatial orientation of the substrates within the reaction chamber to achieve more uniform coating, as well as expanding the range of organosilicon precursors utilized. This approach enables the modulation of the chemical composition and structural characteristics of the resulting films, with a particular focus on the alteration of their wettability and surface properties. This approach will not only improve the reproducibility of single spheroid formation for HMC3 cells but also expand the applicability of the developed platform to other cell types, including primary cultures. This solution is particularly relevant in the context of current trends toward alternative methods aimed at reducing the use of experimental animals [5,6].

## 5. Conclusions

A polymer-like HMDSO coating was deposited using plasma-enhanced chemical vapor deposition at room temperature on Si(100) and PS substrates. For pp-HMDSO film deposited on Si(100), RMS = 1.5 nm, CA = 109.5 ± 0.8°, and SFE = 22.07 mN/m, and for the film deposited on a PS substrate, RMS = 4.52 nm, water CA = 96 ± 3°, and SFE = 26.59 mN/m. These properties can be attributed to the retention of methyl groups (Si–CH_3_) during the coating process. These properties are essential for the weakening of cell adhesion and the initiation of cell–cell interactions.

The results of the biological testing demonstrated that the developed coating effectively induces the formation of compact spheroids from U-87 MG glioblastoma cells. These spheroids are comparable in size and viability to spheroids obtained on commercial ULA plates. In the case of HMC3 microglia cells, the coating efficiency was less pronounced, with the formation of numerous small aggregates being observed. These disparities may be attributable to the biological characteristics of the cell lines and coating inhomogeneity associated with the substrate placement geometry in the PECVD chamber.

The pp-HMDSO coating has emerged as a promising platform for the targeted formation of 3D cellular structures, particularly for cells that are critically dependent on low adhesion. Subsequent research endeavors will center on refining the geometry of sample placement within the PECVD reactor, expanding the range of organosilicon precursors utilized, and manipulating deposition parameters to attain uniform coating across the entire well area. Addressing these challenges will allow the coating to be adapted for a wide range of cell lines, including primary cultures, and will facilitate the introduction of more physiologically relevant in vitro models within the 3R concept (replacement, reduction, and improvement of experimental animal models).

## Figures and Tables

**Table 1:** FTIR band assignment of pp-HMDSO films.

Peak Position (cm^−1^)	Band Assignment
2985	ν_a_(C−H) in CH_3_
2902	ν_s_(C−H) in CH_3_
2130	ν(Si−H)
1408	δ_a_(CH_3_) in Si−CH_3_
1263	δ_s_(CH_3_) in Si−CH_3_
1048	ν_a_(Si−O−Si), ν_a_(Si−O−C)
842	δ(Si-O-Si), ρ(CH_3_) b Si-(CH_3_)_n_, *n* = 3, 2
800	ν(Si−C), δ(Si−O−Si)

a—asymmetric, s—symmetric mode, ν—stretching, δ—bending, ρ—rocking.

**Table 2:** Relative concentrations and atomic ratios of elements in the surface layer of pp-HMDSO film.

Sample	Elemental Composition, at.%			[C]/[Si]	[O]/[Si]	[O]/[C]
	Si	O	C			
pp-HMDSO	25.1	16.3	58.6	2.34	0.65	0.28

**Table 3:** Relative intensities (%) of the Si *2p* peaks, corresponding to silicon in different states, as well as the binding energies of the Si *2p* peaks of the studied sample. The scale is calibrated using the C *1s* line (E_b_ = 284.80 eV).

Sample	Si-C_x_ 101.0 eV	O-Si-C 101.9 eV	SiO_x_ 102.8 eV
pp-HMDSO	22.2	61.9	15.9

**Table 4:** Relative intensities (%) of the C *1s* peaks corresponding to carbon in different states, as well as the binding energies of the C *1s* peaks of the studied sample. The scale is calibrated using the C *1s* line (E_b_ = 284.80 eV).

Sample	C–Si 283.8 eV	C=C 284.8 eV	C–H_x_ 285.7 eV	C–O 286.8 eV
pp-HMDSO	9.0	79.3	8.2	3.5

**Table 5:** The values of the contact angles of pp-HMDSO films.

Samples	CA, °			
	Distilled Water		Diiodomethane	
	Ellipse-Fitting	Young–Laplace	Ellipse-Fitting	Young–Laplace
pp-HMDSO/Si	107 ± 1	109.5 ± 0.8	72 ± 1	73 ± 3
pp-HMDSO/PS	94 ± 3	96 ± 3	-	-

PS—polystyrene. “-”: Diiodomethane contact angles on PS substrates were not determined due to the lack of solvent resistance of PS (swelling/dissolution).

**Table 6:** Values of surface free energy of pp-HMDSO films.

Samples	Neumann Method	Owens–Wendt Method			Wu Method		
	E_s_, mN/m	*E*_s_^d^, mN/m	*E*_s_^p^, mN/m	*E*_s_, mN/m	*E*_s_^d^, mN/m	*E*_s_^p^, mN/m	*E*_s_, mN/m
pp-HMDSO/Si	22.74	22.00	0.07	22.07	24.36	0.30	24.66
pp-HMDSO/PS	26.59	-	-	-	-	-	-

PS—polystyrene. “-” indicates that the Owens–Wendt and Wu method data were not calculated. These methods require the contact angle of diiodomethane on PS, which could not be measured due to solvent incompatibility (swelling/dissolution). Only the total surface free energy (Es) was estimated using the Neumann method.

## Data Availability

The original contributions presented in this study are included in the article/Supplementary Material. Further inquiries can be directed to the corresponding author.

## Supplementary Materials

The following supporting information can be downloaded at: https://www.mdpi.com/article/10.3390/jfb17070334/s1, Figure S1: Surface topography images (25 × 25 µm^2^) of polystyrene plate from AFM; Figure S2: Reference FTIR spectrum of the as-prepared pp-HMDSO film (before storage); Figure S3: Analysis of the surface quality of ULA-plastic reference samples for spheroid formation; Figure S4: The formation of spheroid models from U87MG and HMC3 cells has been observed on experimental plates; Figure S5: The formation of spheroids from 2500 and 5000 U-87 MG and HMC3 and cell viability additional samples using pp-HMDSO-coated plastic plates; Figure S6: Comparison of the volumes of spheroidal models in the control and experimental plate; Figure S7: Light microscopy image (10× magnification, scale bar = 200 μm) showing the bottom of an untreated U-shaped well of a 96-well plate.

## Abbreviations

The following abbreviations are used in this manuscript:

AFM	Atomic Force Microscopy
ATCC	American Type Culture Collection
BME	Basement Membrane Extract
BSA	Bovine Serum Albumin
CA	Contact Angle
DMEM/F12	Dulbecco’s Modified Eagle Medium/Nutrient Mixture F-12
EDS	Energy Dispersive Spectroscopy
EGF	Epidermal Growth Factor
FDA	Fluorescein Diacetate
FBS	Fetal Bovine Serum
FTIR	Fourier Transform Infrared Spectroscopy
GelMA	Gelatin Methacryloyl
HMDSO	Hexamethyldisiloxane
HMC3	Human Microglia Clone 3
MDCK	Madin–Darby Canine Kidney
NFC	Nanofibrillar Cellulose
PBS	Phosphate-Buffered Saline
PEG	Polyethylene Glycol
PECVD	Plasma-Enhanced Chemical Vapor Deposition
PHEMA	Poly(2-hydroxyethyl methacrylate)
PI	Propidium Iodide
PLA	Polylactic Acid
PLCL	Poly(L-lactide-co-ε-caprolactone)
PMEA	Poly(2-methoxyethyl acrylate)
pp-HMDSO	Plasma-polymerized Hexamethyldisiloxane
PS	Polystyrene
PVA	Polyvinyl Alcohol
RF	Radio Frequency
RMS	Root Mean Square (Roughness)
SEM	Scanning Electron Microscopy
SE	Standard Error of the Mean
SFE	Surface Free Energy
Si(100)	Silicon (100) Substrate
SiCOH	Silicon Oxycarbide
STR	Short Tandem Repeat
TCP	Tissue Culture Polystyrene
ULA	Ultra-Low Attachment
U-87 MG	Human Glioblastoma Cell Line
XPS	X-ray Photoelectron Spectroscopy
α-MEM	Alpha Minimum Essential Medium
3D	Three-dimensional
2D	Two-dimensional
