# Supplementary material for: Hydrophobic pp-HMDSO Coating for Three-Dimensional Cell Culture
Source: J Funct Biomater. 2026 Jul 9;17(7):334. doi: 10.3390/jfb17070334 (PMC13412863; doi:10.3390/jfb17070334)
Supplement: Supplementary file 1 [file jfb-17-00334-s001.zip › jfb-4291478-supplementary.pdf]

Supplementary material

The figures below present additional data that supplement the experiments described in the main text. They include AFM images of both untreated and plasma-treated polystyrene surfaces, which show the coating morphology directly on the culture substrate; a reference FTIR spectrum of the freshly deposited pp-HMDSO film; control images demonstrating spheroid formation on commercial ULA plates and on untreated plastic; replicate experiments on pp-HMDSO-coated plates; further examples of spheroid formation and cell viability on coated substrates; a quantitative comparison of spheroid volumes across all tested plate types; and an image showing the shape of an untreated well to illustrate the well geometry. Each figure is cited in the main text and is included to provide a fuller picture of the experimental data without making the main figures overly crowded.

(a) (b)

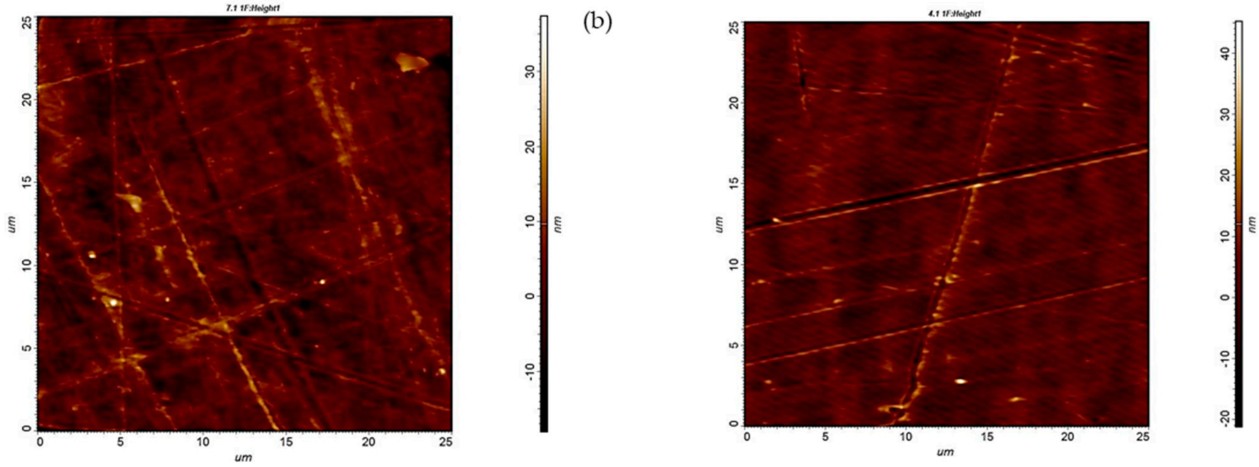

Figure S1. Surface topography images (25 × 25 μm²) of polystyrene plate from AFM: (a) untreated sample and (b) air-plasma-treated sample. The images show the surface morphology of the actual culture substrate before and after plasma treatment.

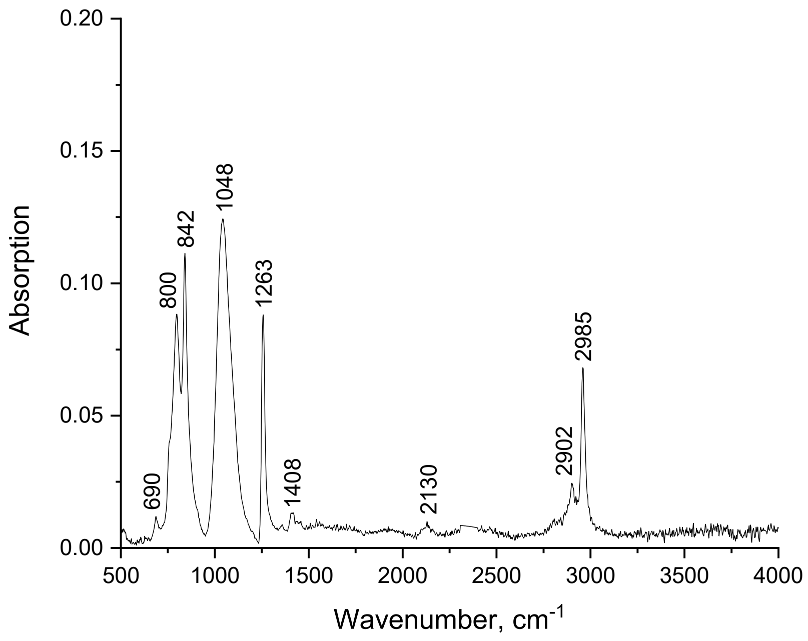

Figure S2. Reference FTIR spectrum of the as-prepared pp-HMDSO film (before storage). The characteristic bands of Si–O–Si (1048 cm^{-1}), Si–CH_3 (1263 cm^{-1}) and Si–H (2130 cm^{-1}) are marked. This spectrum corresponds to the "as-grown" reference in Figure 5 of the main text. The FTIR spectrum of pp-HMDSO film.

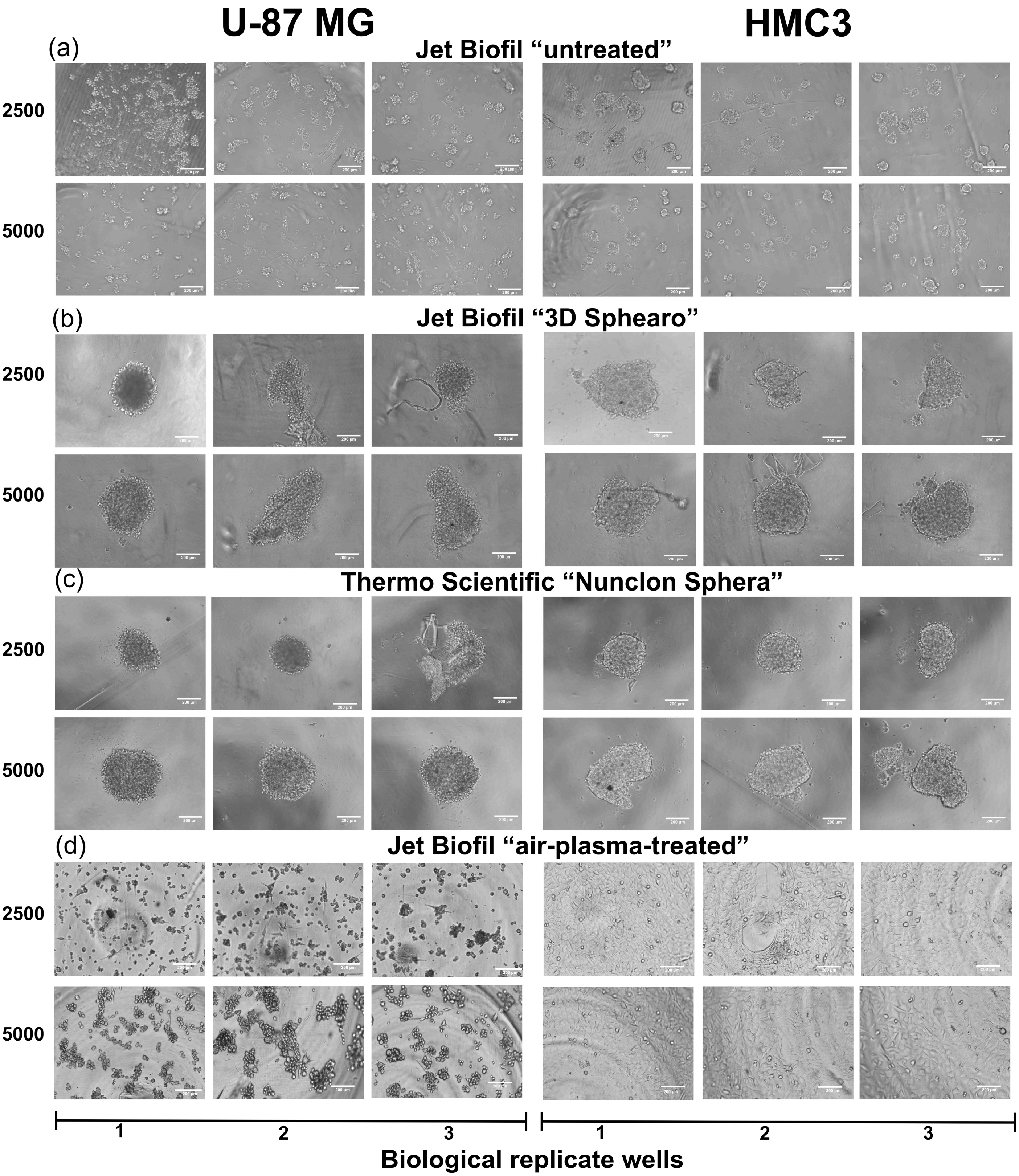

Figure S3. Analysis of the surface quality of ULA-plastic reference samples for spheroid formation: (a) negative control: untreated polystyrene (JET Biofil untreated); **(b)** and **(c)** – positive commercial controls: **(b)** 3D Sphearo™ Ultra-low Adsorption Surface (JETBiofil); **(c)** Nunclon™ Sphera™ (Thermo Fisher Scientific); **(d)** additional control to assess the effect of the plasma itself**:** treated by air-plasma-only (air-plasma-treated, JETBiofil). The formation of spherical structures from U-87 MG and HMC3 cells following a one-day culture period is observed. The specimen was observed under light microscopy with 10x magnification, scale bar 200 μm. These controls confirm

that spheroids form only on commercial ULA plates, not on untreated or plasma-only treated plastic.

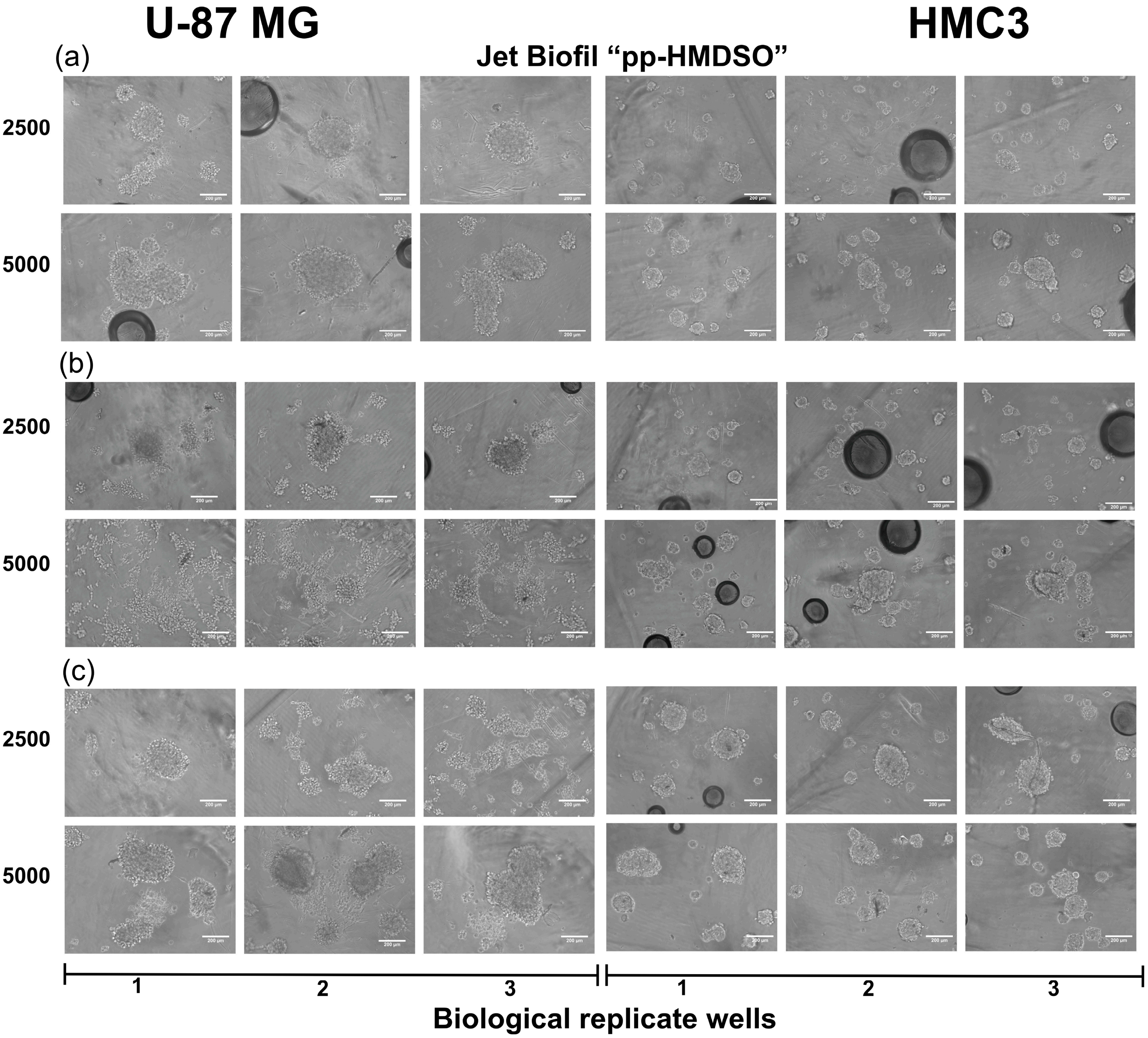

Figure S4. **The formation of spheroid models from U87MG and HMC3 cells has been observed on experimental plates.** Biological replicate wells of a 96-well plastic (n=3) sample plate with pp-HMDSO: **(a)** sample 1; **(b)** sample 2; **(c)** sample 3. The formation of spherical structures from U-87 MG and HMC3 cells following a one-day culture period is observed. The specimen was observed under light microscopy with 10x magnification, scale bar 200 μm. These images supplement Figure 9 and demonstrate the reproducibility of the coating performance across three independent samples.

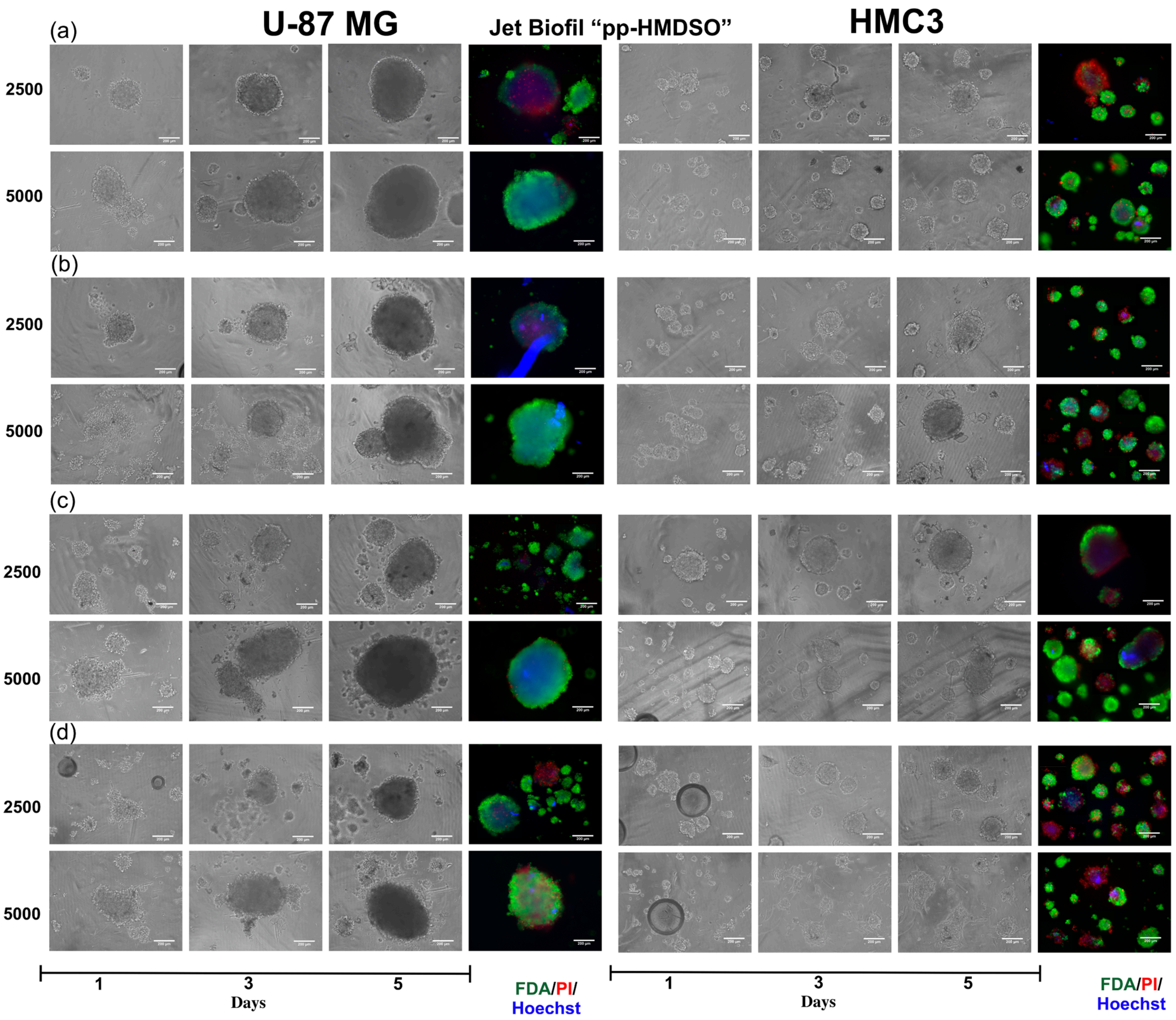

Figure S5. **The formation of spheroids from 2,500 and 5,000 U-87 MG and HMC3 and cell viability additional samples using pp-HMDSO-coated plastic plates.** Samples plate with pp-HMDSO: **(a)** sample 4; **(b)** sample 5; **(c)** sample 6; **(d)** sample 7. The green signal indicates viable cells, the red signal indicates dead cells, and the blue signal indicates the total cell number. Light and fluorescence microscopy were utilized, with a magnification of 10x, scale bar 200 μm. These data further confirm the consistency of the coating performance across multiple independent fragments.

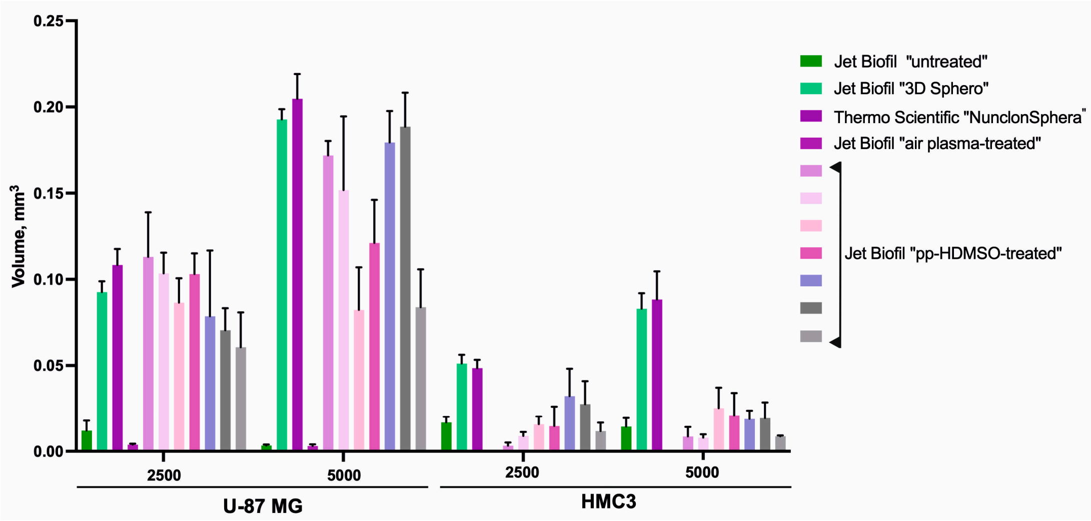

Figure S6. **Comparison of the volumes of spheroidal models in the control and experimental plate:** negative control: untreated polystyrene (JET Biofil untreated); positive commercial controls: 3D Spheаro™ Ultra-low Adsorption Surface (JETBiofil) and Nunclon™ Sphera™ (Thermo Fisher Scientific); additional control to assess the effect of the plasma itself: treated by air-plasma-only (air-plasma-treated, JETBiofil); samples of experimental plate pp-HMDSO-coated, (JETBiofil). The quantity of U-87 MG and HMC3 spheroids on day 5 of culture is contingent upon the type of culture medium employed. The results are expressed as the mean ± standard error. This figure supplements the quantitative analysis presented in Figure 10

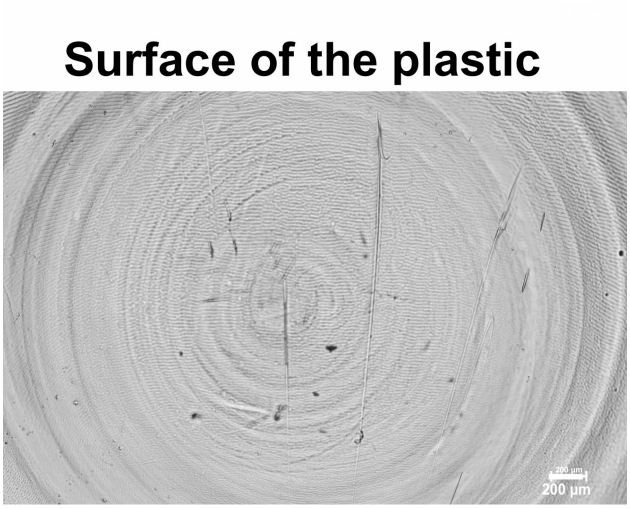

Figure S7. Light microscopy image (10× magnification, scale bar = 200 µm) showing the bottom of an untreated U-shaped well of a 96-well plate. The image illustrates the typical concave geometry of the well and reveals minor surface irregularities commonly present on the moulded polystyrene surface of such plates. These features are shown for reference, as they represent the actual substrate used in the control experiments.
